# The Effects of Virtual Reality on Hope and Travel Expectations in Healthy and Hospitalized Children: Quasi-Experimental Design Approach

**DOI:** 10.2196/65311

**Published:** 2025-06-16

**Authors:** Pei-Shan Hsieh, Hsiu-Sen Chiang, Fang-Liang Huang, Wen-Hsin Chi

**Affiliations:** 1 Department of Intelligent Commerce, National Kaohsiung University of Science and Technology, No. 58, Shenzhong Rd, Yanchao Dist, Kaohsiung City, 824004, Taiwan, 886 7-3814526 ext 17528, 886 7-3814526; 2 Department of Information Management, National Taichung University of Science, Taichung City, Taiwan; 3 Department of Children’s Medical Center, Taichung Veterans General Hospital, Taichung, Taiwan; 4 Institute of Management of Technology, National Yang Ming Chiao Tung University, Hsinchu, Taiwan

**Keywords:** hospitalized children, virtual reality, ECG, hope, travel expectations, electrocardiogram

## Abstract

**Background:**

Virtual reality (VR) has become a powerful tool for enhancing the experiences of patients with critical illnesses, particularly hospitalized children with leukemia. Since traveling is nearly impossible for them, St Jude has teamed up with the travel company Expedia to launch “Dream Adventures,” a pilot program that offers immersive VR experiences, allowing children to explore new destinations from the comfort of the hospital.

**Objective:**

The aim of this study was to evaluate the pleasurable experience of VR and its impact on healthy and hospitalized children’s travel expectations and hope by using electrocardiography (ECG) and questionnaires to enhance research objectivity.

**Methods:**

Participants were children aged 7-18 years, divided into 2 distinct groups: 30 healthy children and 18 hospitalized children with leukemia. Both groups received the same VR intervention and were assessed using a 1-group pretest-posttest design. The questionnaires were designed to assess differences in the children’s sense of hope and travel expectations, and their physiological data were collected through ECG.

**Results:**

The results indicated a statistically significant increase in hope levels from pre-VR to post-VR intervention in both healthy children (preintervention: mean 5.83, SD 0.87; postintervention: mean 6.36, SD 0.76; *P*=.002) and hospitalized children (preintervention: mean 5.51, SD 1.17; postintervention: mean 5.73, SD 1.15; *P*=.03), as determined by paired samples 2-tailed *t* tests. Furthermore, an independent samples 2-tailed *t* test revealed a significant difference in postintervention hope levels between the hospitalized children (mean 5.73, SD 1.15) and healthy children (mean 6.36, SD 0.76; *P*=.05). Then, we further compared the mean differences in hope scores from preintervention to postintervention. Healthy children demonstrated a greater increase (an increase of 0.53, from 5.83 to 6.36) than the hospitalized children (an increase of 0.22, from 5.51 to 5.73). In terms of physiological responses, ECG indicators such as SD of all normal-to-normal intervals and low-frequency power revealed significant differences in autonomic nervous system activity between the 2 groups. Healthy children exhibited higher sympathetic activation, suggesting greater emotional engagement during the VR experience, whereas hospitalized children demonstrated more attenuated responses. The consistency between physiological data and self-reported measures strengthens the construct validity of the instruments used and enhances the overall reliability of the study findings.

**Conclusions:**

The VR intervention significantly increased hope levels in both healthy children and hospitalized children with leukemia, with a greater improvement observed among healthy participants. Therefore, this study suggests that when designing interventions for hospitalized children, more targeted emotional support strategies should be considered. Future studies are recommended to explore different types of VR content and the medical conditions of hospitalized children.

## Introduction

Nowadays, virtual reality (VR) has gradually become a powerful tool for enhancing the patient experience in children’s hospitals [1]. Immersive experiences allow individuals to fully engage in realistic environments created through digital technologies or designed settings, fostering deep emotional connections [2]. VR apps initially started with simple graphics for entertainment and research but are now widely used across many professional fields [3]. As VR technology continues to advance, its apps in the medical field are gaining increasing recognition. For instance, VR is being used in various medical domains [4], including medical education [5,6], clinical decision-making [7,8], preoperative planning [9], patient education [10,11], and surgical simulation [12].

As part of VR content creation, 360-degree video is an innovative medium that captures a scene from all directions using specialized cameras, allowing users to explore the environment as if they were physically present [13]. The sense of spatial presence created by 360-degree videos positions them as a form of immersive VR experience [14,15], particularly due to their ability to evoke strong emotions and make users feel surrounded by the content [16,17]. Previous studies in nonhospital settings have shown that VR has significant potential, as it can reduce psychological distress in young people across various situations [18-20] and elicit varying degrees of physical activity intensity in young healthy adults [21].

Additionally, there is a great potential for VR to address the unique medical care needs of hospitalized children [22]. For example, for children battling serious illnesses at St Jude Children’s Research Hospital, traveling may seem out of reach. To bring the world to these young patients, St Jude has teamed up with the travel company Expedia to launch “Dream Adventures,” a pilot program that offers immersive VR experiences, allowing kids to explore new destinations from the comfort of the hospital [23].

Mitsea et al [24] highlighted that VR-based positive mental imagery training, when combined with reward training, effectively reduced anhedonia while increasing pleasure and self-motivation skills. van Gelderen et al [25] implemented VR-based Eye Movement Desensitization and Reprocessing therapy, where patients walked on a treadmill while engaging with personal trauma-related images and relaxing music. The results indicated that such interventions could effectively reduce feelings of isolation and foster hope. Additionally, Bush et al [26] found that the Virtual Hope Box application enhanced users’ hope and emotional regulation skills. Therefore, the aim of our study was to examine the effects of VR on pleasure, hope, and travel expectations in hospitalized children with leukemia and further compare them with healthy children.

Researchers have used videos to elicit specific emotions and collect emotion-related data. More recently, VR has been used to evoke particular emotional responses [27]. VR offers a more objective method for eliciting emotions by minimizing environmental interferences [28]. However, health care providers have perceived certain barriers to using VR, such as concerns about older adults with cognitive impairments [29]. Nevertheless, studies have suggested that VR apps are feasible for use in hospital wards and do not cause significant side effects [30,31]. Self-reported emotion measurements are susceptible to recall bias and cultural influences, whereas electrocardiography (ECG) provides a reliable, real-time evaluation of emotional variations.

Recent studies have demonstrated that ECG is a viable signal for measuring stress [32], and research in the study by Schmidt et al [33] has shown the feasibility of ECG-based stress assessment in a VR environment. Ahmad et al [34] collected ECG data from 9 participants experiencing a VR roller coaster. von Rosenberg et al [35] investigated the impact of mental stress on physiological responses using a wireless ECG. Furthermore, Kalatzis et al [36] examined whether VR scenarios could elicit specific affective states and developed a validated database to predict emotional states (eg, calm vs acute stress) using ECG. Additionally, Scherz et al [2] used VR as a significant stressor, analyzing heart rate and other physiological parameters through ECG. Their results indicated that while using a VR headset influenced heart rate and the root-mean-square of the successive differences in heartbeats, it did not significantly increase driving-related stress.

Therefore, the aim of this study was to evaluate the pleasurable experience of VR and its impact on hospitalized children’s travel expectations and sense of hope by using ECG and questionnaires approach. This study incorporated the collection of physiological data through ECG to analyze and corroborate emotional changes in children based on physiological signals.

## Methods

### Study Design and Setting

This study used a quasi-experimental design to investigate the effects of VR on hope, pleasure, and travel expectations in children. The study included 2 distinct groups of participants: healthy children and hospitalized children with leukemia. Both groups received the same VR intervention and were assessed using a 1-group pretest-posttest design, in which outcome measures were collected immediately before and after the intervention within each group. This design allowed us to examine within-group changes in hope, pleasure, and travel expectations, as well as to compare the differential effects of the VR intervention between the 2 populations.

### Participants

The experiment was conducted in 2 stages: a pilot test and a formal test. The purpose of the pilot test was to determine the VR destination. Five domestic tourist destinations (Park Lane by China Metal Products, National Museum of Natural Science, Dadu Blue Highway, Shalu Dream Street, and Dakeng Pink Valentine Bridge) and 6 international tourist destinations (the Pyramids of Egypt, the Eiffel Tower in France, Neuschwanstein Castle in Germany, Venice in Italy, Aphrodisias in Turkey, and the Space Station) were provided as options. Based on the results of the pilot test involving 12 children aged 7 -18 years, the Eiffel Tower was selected as the VR content due to its high ratings for engagement and recognizability. The content consisted of 8 scenes of the tower, captured from various angles and at different times of day, and participants were able to switch between scenes freely using the VR device.

In the formal test, participants were included 2 distinct groups of participants: healthy children and hospitalized children with leukemia. The hospitalized children with leukemia were recruited from the pediatric ward of Taichung Veterans General Hospital in Taiwan based on inclusion and exclusion criteria recommended by the attending physician. These children often undergo strict regimens of chemotherapy, radiation, or other therapies, which limit their opportunities to travel and experience new places, particularly if they have spent significant time in the hospital. This context likely heightens their desire to engage in virtual travel experiences, making them a key focus of this study. Additionally, the healthy children were recruited from a local cram school, where they were enrolled in regular academic programs and had no known history of serious medical conditions. Recruitment procedures prioritized voluntary participation. The inclusion and exclusion criteria of formal experimental design are summarized in Textbox 1.

Textbox 1. **Inclusion and exclusion criteria for participants in the formal study design.**

Inclusion criteria

- Hospitalized children: hospitalized children with leukemia aged 7-18 years, in the midstage of treatment with stable vital signs.
- Healthy children: healthy children aged 7-18 years.

Exclusion criteria

- Hospitalized children: children unable to independently use virtual reality equipment or those diagnosed with epilepsy.
- Healthy children: children unable to independently use virtual reality equipment.

### Ethical Considerations

All procedures performed in studies involving human participants adhered to the ethical standards of the institutional and national research committee, as well as the 1964 Declaration of Helsinki and its later amendments or comparable ethical standards.

This study was approved by the Institutional Review Board of Taichung Veterans General Hospital (CF20285A) on December 3, 2020, and was conducted from December 3, 2020, to June 30, 2021. Informed consent was obtained from all participants and their parents or guardians prior to the study, with signed consent forms collected. The consent process involved clearly communicating the study’s objectives, procedures, and potential risks to ensure voluntary participation. All data were anonymized to protect participants’ privacy.

To show appreciation for participants’ involvement, each participant will receive a VR cardboard viewer (valued at approximately US $1.50) upon completing the experiment. This compensation allows them to continue experiencing VR content after the study. It does not affect their right to participate voluntarily or withdraw from the study at any time.

### Instruments

This study adopted the pleasure measurement from the study by Bakhuis et al [37]. A higher score indicated that the subject experienced more pleasure from the stimulus as shown in Table 1.

**Table 1.** Scale items used to measure pleasure in response to virtual reality stimuli^a^.

Items	Scores
1. Unhappy to happy	1‐7
2. Unsatisfied to satisfied	1‐7
3. Melancholic to cheerful	1‐7
4. Despairing to hopeful	1‐7
5. Bored to surprised	1‐7

a From Schifferstein and Tanudjaja [38].

In addition, we measured children’s hope using the scale developed by Snyder et al [39]. The scale was scored on a 7-point Likert scale, with responses ranging from 1 (“definitely disagree”) to 7 (“definitely agree”). Travel expectations in this study were based on the anticipated outcomes a person expects to gain while traveling to a destination [40]. The items measuring children’s hope and travel expectations are listed in Textbox 2.

Textbox 2. **Scale items for measuring children’s hope and travel expectations.**

Items of measuring children’s hope [39]:

1. My life is very good now.
2. I can think of many ways to get the things in life that are most important to me.
3. I am doing just as well as other kids of my age.
4. I have a lot of confidence in myself.
5. When I have a problem, I can come up with lots of ways to solve it.
6. I think the things I have done in the past will help me in the future.
7. Even when others want to quit, I know that I can find ways to solve the problem.
8. I think my life is full of hope.

Items of measuring travel expectations [40]:

1. I can learn new knowledge.
2. I can learn new culture.
3. I can experience different things.
4. I can see the beautiful view.

### Procedure

**Figure 1.**
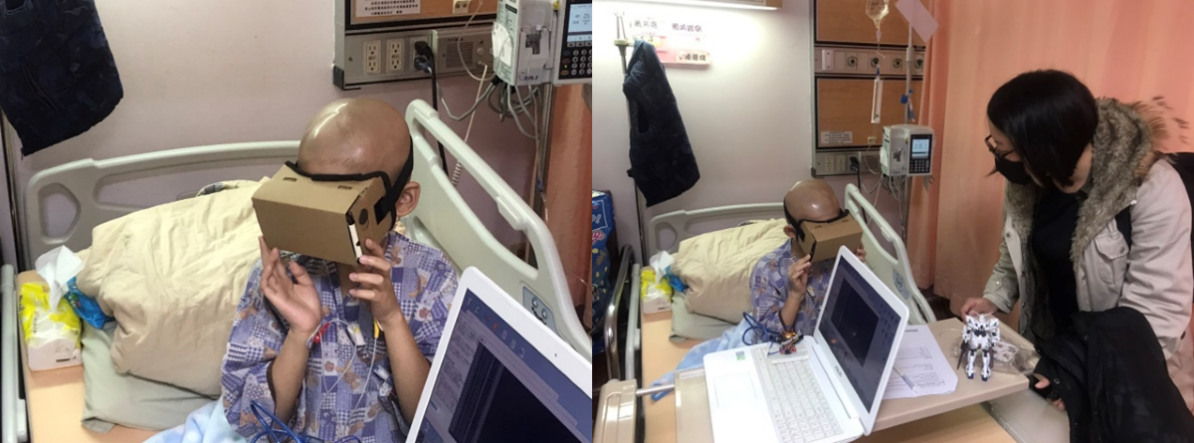
Hospitalized children were tested in a hospital ward bed.

The researcher tested 1 child at a time, with each session lasting approximately 40 minutes. Healthy children were tested individually in a classroom, while hospitalized children were tested in their hospital ward beds, as shown in Figure 1. The study prioritized participant safety during VR use. Potential risks associated with VR, such as motion sickness, visual discomfort, or dizziness, were considered during the design phase. To mitigate these risks, age-appropriate 360-degree VR content with calming visuals was selected. Participants were monitored throughout the session, and breaks were allowed if discomfort occurred.

As the participants were underage and part of a vulnerable group, the attending physician and researcher carefully explained the experiment and obtained consent from both the children and their parents. The study followed a structured procedure: (1) Collecting children’s resting heart rate for baseline ECG data collection, followed by completing the pretest questionnaire to measure hope and travel expectations. (2) An 11-minute VR experience while ECG data were recorded. (3) Completing the posttest questionnaire to measure hope, travel expectations, and pleasure outcomes.

The experiment was conducted individually in classrooms (healthy children) or hospital wards (hospitalized children). .

### Data Analysis

All scales were tested using SPSS software (version 20; IBM Corp). We assessed the reliability of the items by examining the Cronbach alpha values, all of which exceeded the common threshold of 0.70, suggesting adequate reliability [41]. The pleasure scale demonstrated strong internal consistency, with a Cronbach α value of 0.88. Similarly, the hope scale exhibited high reliability, with a Cronbach α value of 0.93. Additionally, the travel expectations scale showed adequate internal consistency, with a Cronbach α value of 0.73. To analyze the data related to hope, travel expectations, and pleasure—key factors for a successful VR intervention—the questionnaire findings were compared between groups using an independent 2-tailed *t* test. Additionally, post hoc analyses were conducted using MANOVA to examine differences between children who had traveled (once or more) and those who had not. Independent samples 2-tailed *t* tests were also performed to compare children with and with no prior VR experience.

An ECG typically requires a physiological signal length of 30 seconds to 5 minutes for heartbeat detection [42]; therefore, this study used 5 minutes as the benchmark. To extract the R-peak of the QRS complex (the QRS complex is the portion of the ECG that reflects the depolarization of the ventricles, marking the onset of ventricular contraction; it is composed of the Q, R, and S waves and is a key indicator of cardiac electrical activity), we applied the Pan-Tompkins open-source algorithm using MATLAB [43]. During the process of collecting physiological signals, the original signal is prone to noise interference, making the QRS complex of the ECG difficult to distinguish. To address this, a filter was used to remove unnecessary signals, enhancing the ECG waveform’s characteristics.

**Figure 2.**
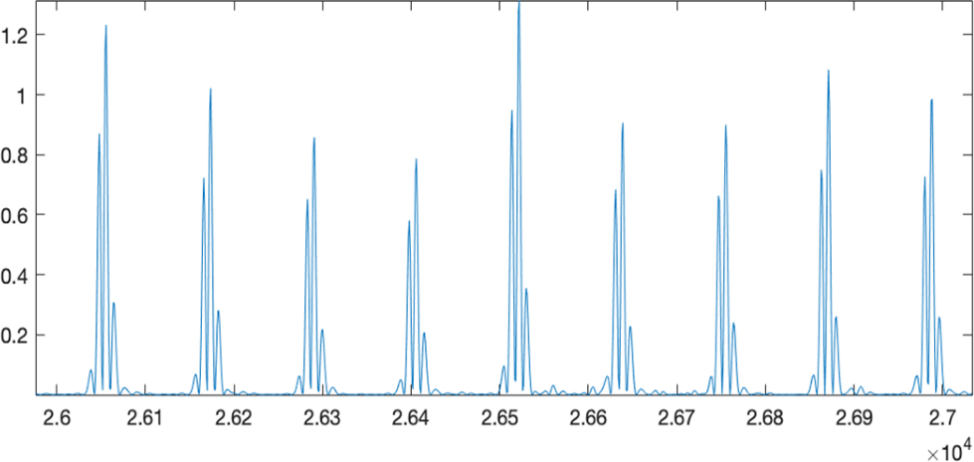
Actual physiological signal after square.

Since heart rate variability is calculated based on the highest peak (R-peak) in the ECG, a Butterworth high-pass filter was applied to eliminate unwanted frequency bands and highlight the R-peak within the QRS complex. To detect the position of the R-peak, the differentiated signal was first segmented. Then, squaring was applied to amplify the signal, making high-frequency values more prominent, as shown in Figure 2.

Finally, the moving window integral method was used, with 30 samples (approximately 150 ms) as the window size. When both the difference threshold and the integration threshold met the required conditions simultaneously, an R-peak was detected, and the threshold was automatically updated.

**Figure 3.**
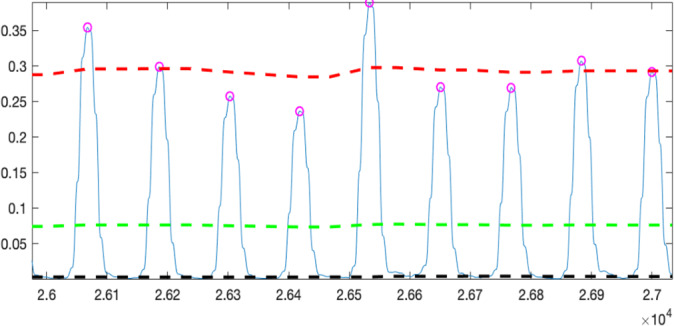
Actual physiological signal results after moving window integral.

**Figure 4.**
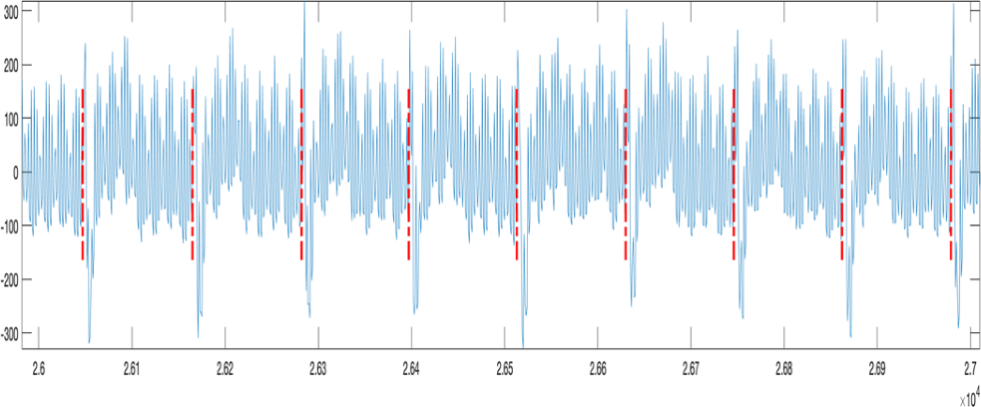
R-peak pulse sequence diagram.

As shown in Figure 3, the black line represents the noise threshold curve, the green line represents the automatically adapted threshold curve, and the red line represents the predicted R-peak curve. Finally, the results of the R-peak sequence were used to plot the pulse sequence onto the original waveform, as shown in Figure 4, which can be used to evaluate accuracy.

## Results

### Sample Characteristics

The participants included 18 hospitalized children and 30 healthy children, all aged between 7 and 18 years (see Table 2 for details). The hospitalized children were preselected by the attending physician and were patients with leukemia with stable health who were willing to participate in the experiment.

**Table 2.** Descriptive statistics of participants in the formal test by group.

	Hospitalized children (n=18) , n (%)	Healthy children (n=30), n (%)
Gender		
Boy	11 (61)	23 (77)
Girl	7 (39)	7 (23)
Age (years)		
7‐9	6 (33)	N/A^a^
10‐12	3 (17)	18 (60)
13‐15	6 (33)	12 (40)
16‐18	3 (17)	N/A
Experience of traveling abroad		
0 times	8 (45)	11 (37)
1‐5 times	6 (33)	17 (57)
5‐10 or >10 times	4 (22)	2 (6)
Experience of using VR^b^		
Used	8 (44)	9 (30)
Never used	10 (56)	21 (70)

a N/A: not applicable.

b VR: virtual reality.

We use the MANOVA test for post hoc analyses to compare 2 different dependent variables (hope and travel expectations) among 3 groups based on their experience of traveling abroad (0 times, 1‐5 times, and more than 10 times) in the hospitalized children. The output shows that there are no significant differences in hope among groups (experience of traveling abroad 0 times: mean 5.34, SD 1.15; 1‐5 times: mean 5.44, SD 1.11; *P*=.99; and 0 times: mean 5.34, SD 1.15; more than 10 times: mean 5.34, SD 1.40; *P*>.99). Similarly, there are no significant differences in travel expectations among groups (experience of traveling abroad 0 times: mean 6.14, SD 0.85; 1‐5 times: mean 5.75, SD 1.00; *P*=.70; and 0 times: mean 6.14, SD 0.85; 5‐10 times: mean 6.31, SD 0.94; *P*=.94). Also, the results show that there are no significant differences in hope among groups in the healthy children.

In addition, we adopt an independent 2-tailed *t* test to analyze 2 different dependent variables (hope and travel expectations) between 2 groups based on their experience of using VR (used and never used) in hospitalized and healthy children, respectively. In the hospitalized children group, the results show that there are no significant differences in hope between the 2 groups (experience of using VR: used vs never used; *t*=0.57; *P*=.58), and no significant differences in travel expectations between the 2 groups (*t*_17_=−0.04; *P*=.97). Similarly, the results show that there are no significant differences in hope and travel expectations in the healthy children group.

### Hope, Pleasure, and Travel Expectations Results

First, there was no significant difference in the sense of hope between the hospitalized and healthy children before using VR (*t*_46_=−1.02; *P*=.32). However, after using VR to experience tourist attractions, a significant difference was observed between the 2 groups (*t*_46_=−2.06; *P*=.049) (Table 3).

**Table 3.** Levels of hope before and after watching virtual reality in the hospitalized and healthy children during the formal test^a^.

Stage and group	Mean (SD)	*t* test (*df*)	*P* value^b^
Before watching VR^c^		−1.02 (46)	.32
Hospitalized children	5.51 (1.17)		
Healthy children	5.83 (0.87)		
After watching VR		−2.06 (46)	.049
Hospitalized children	5.73 (1.15)		
Healthy children	6.36 (0.76)		

a An independent samples 2-tailed *t* test was conducted to compare levels of hope between the hospitalized children and the healthy children before and after watching VR.

b Significance at .05 level.

c VR: virtual reality.

To further examine the degree of improvement in hope after the experiment, we conducted paired samples 2-tailed *t* tests for each group separately. The results showed significant differences in both the hospitalized children group (*t*_17_=−2.46; *P*=.03) and the healthy children group (*t*_29_=−3.46; *P*=.002). Furthermore, an independent samples 2-tailed *t* test revealed a significant difference in postintervention hope levels between the hospitalized and healthy children (*t*_46_=−2.06; *P*=.049). Then, we further compared the mean differences in hope scores from preintervention to postintervention using an independent samples 2-tailed *t* test and found that the difference was statistically significant (*t*_46_=2.18; *P*=.03). Healthy children demonstrated a greater increase in mean hope scores (an increase of 0.53, from 5.83 to 6.36) compared with the hospitalized children (an increase of 0.22, from 5.51 to 5.73). This suggest that using VR to experience tourist attractions may be more effective in enhancing the sense of hope in healthy children (Table 4). On the other hand, this result may also indicate that healthy children’s sense of hope is more easily stimulated than that of hospitalized children.

**Table 4.** Comparison of hope levels before and after watching virtual reality within the hospitalized and healthy children during the formal test^a^.

Group and stage		Mean (SD)		*t* test (*df*)	*P* value^b^
Hospitalized children				−2.46 (17)	.03
	Before watching VR^c^	5.51 (1.17)			
	After watching VR	5.73 (1.15)			
Healthy children				−3.46 (29)	.002
	Before watching VR	5.83 (0.865)			
	After watching VR	6.36 (0.756)			

a Paired samples 2-tailed *t* tests were conducted separately within the hospitalized children and healthy children to examine the change in hope levels before and after watching VR.

b Significance at .05 level.

c VR: virtual reality.

In addition, the results of the independent samples 2-tailed *t* test revealed a nonsignificant difference in pleasure (*t*_46_=−1.116; *P*=.27) and travel expectations (*t*_46_=−0.300; *P*=.77) between the hospitalized and healthy children. We inferred that both hospitalized and healthy children experienced pleasure when using VR, likely because it was a novel experience for them, and they had expectations about traveling.

### Power and Effect Size for Independent Samples 2-Tailed *t* Test

For the independent samples 2-tailed *t* test, Cohen *d* is calculated by taking the mean difference between the 2 groups and dividing it by the pooled SD. Cohen *d* is an appropriate effect size measure when both groups have similar SDs and equal sample sizes. However, since our sample sizes differ, we used Hedges *g*, which adjusts for sample size differences. In this study, Hedges *g* was calculated as:


$$\text{Hedges }g=\frac{\left(6.4-5.7\right)}{0.967291}=0.7$$

According to Zach [44], a *g* value of 0.2 represents a small effect size, 0.5 represents a medium effect size, and 0.8 represents a large effect size. Therefore, the effect size in this study (0.7) is close to a large effect size.

### ECG Data Analysis Result

The resting state represents the baseline heartbeat. In this study, it was used to determine whether the heart rates of the 2 groups (hospitalized children and healthy children) were at the same level before the experiment. The results showed no significant difference between the groups before using VR.

However, when using VR, 5 indicators showed significant differences between the 2 groups: mean normal-to-normal intervals (*t*_46_=−2.119; *P*=.04), normal-to-normal intervals (*t*_46_=−2.856; *P*=.007), SD of normal-to-normal intervals (SDNN; *t*_46_=2.189; *P*=.04), low-frequency (LF; *t*_46_=2.834; *P*=.008), and high-frequency normalized units (HF_nu;*t*_46_=2.297; *P*=.03). The results are summarized in Table 5.

**Table 5.** Differences in electrocardiography indicators between the hospitalized and healthy children while watching virtual reality^a^.

Autonomic nervous system, indicator, and group			Mean (SD)		*t* test (*df*)	*P* value^b^
Parasympathetic nerves						
	SDNN^c^				2.19 (46)	.04
		Group 1^d^	150.08 (43.75)			
		Group 2^e^	119.12 (38.59)			
	HF_nu^f^				2.30 (46)	.03
		Group 1	74.82 (15.32)			
		Group 2	64.38 (11.03)			
Sympathetic nerve						
	LF^g^				−2.83 (46)	.008
		Group 1	192.77 (220.48)			
		Group 2	443.64 (307.65)			

a This table shows the results of independent samples 2-tailed *t* tests comparing electrocardiography indicators between hospitalized children and healthy children during virtual reality viewing.

b Significance at .05 level.

c SDNN: SD of all normal-to-normal intervals.

d Group 1: hospitalized children.

e Group 2: healthy children.

f HF_nu: high-frequency normalized units.

g LF: low-frequency power.

In the ECG analysis, index calculations can be performed after obtaining the R-R interval. According to the literature, the autonomic nervous system (ANS) is mainly composed of the sympathetic nervous system (SNS) and the parasympathetic nervous system (PNS). If the ANS is biased toward the SNS, the PNS has a smaller effect. To illustrate, if the ANS were compared with a car, the SNS would represent the accelerator, and the PNS would represent the brake.

ECG indicators, such as SDNN and HF_nu, are related to the PNS, while LF is related to the SNS. In this study, the mean value of the SNS indicator (LF) in the hospitalized children (mean 192.77, SD 220.48) was significantly lower than the mean value of the SNS indicator (LF) in the healthy children (mean 443.64, SD 307.65). Conversely, the mean values of parasympathetic nervous indicators (SDNN, HF_nu) were significantly higher in the hospitalized children than those in the healthy children. Based on the significant results from LF, it appears that the SNS is highly active during VR exposure, indicating that healthy children experience VR in a more excited state. The results of the formal test are summarized in Table 6.

**Table 6.** Summary of formal test results comparing the hospitalized and healthy children across physiological and psychological measures.

Measurement and results		Significance
ECG**^a^**		
	Comparison of the ECG indicators of G1^b^ and G2^c^ when in a resting state.	Not significant
	Comparison of the ECG indicators of G1 and G2 when using VR^d^.	Significant
Hope		
	Comparison of the state of hope in G1 and G2 before using VR.	Not significant (*P*=.32)
	Comparison of the state of hope in G1 and G2 after using VR.	Significant (*P*=.05)
	Improvement of G1’s state of hope.	Significant (*P*=.03); the mean value rose by 0.22, from 5.51 to 5.73.
	Improvement of G2’s state of hope.	Significant (*P*=.002); the mean value rose by 0.53, from 5.83 to 6.36.
Pleasure		Not significant (*P*=.27)
Travel expectations		Not significant (*P*=.77)

a ECG: electrocardiography.

b G1: hospitalized children with leukemia.

c G2: healthy children.

d VR: virtual reality.

## Discussion

### Summary of Key Findings

We assessed the effects of VR on hope, pleasure, and travel expectations in healthy and hospitalized children using ECG and questionnaires. The study demonstrated consistency between self-reported data and physiological measures, suggesting that VR effectively enhanced hope among both healthy and hospitalized children with leukemia, with a more pronounced effect in the healthy group. However, no significant changes were observed in pleasure or travel expectations in either group.

### Clinical and Research Implications

While questionnaires captured subjective experiences, ECG objectively measured real-time physiological responses. Indicators such as SDNN and LF revealed differences in ANS activity. Healthy children exhibited higher sympathetic activation, reflecting greater engagement, while hospitalized children showed more subdued responses. This alignment validates the questionnaire measures and enhances the study’s reliability. Beyond validation, ECG plays a crucial role in detecting subtle or unconscious emotional responses and tracking real-time fluctuations during VR exposure. By linking subjective perception with objective data, ECG strengthens methodological rigor and supports future VR-based emotional and physiological research.

We infer that this result possibly reflects hospitalized children’s deeper psychological needs and the greater impact of long-term hospitalization stress. Their sense of hope may be more focused on overcoming illness and achieving milestone progress in treatment. It is likely linked to improvements in their health condition, disease remission, or the possibility of participating in activities they have missed due to their illness. Therefore, this suggests that when designing interventions for hospitalized children, more targeted emotional support strategies should be considered. This aligns with previous research, in which Napetschnig et al [45] proposed that promoting user motivation is one of the key quality criteria for developing user-centered VR.

Nevertheless, we found a significant difference in the mean hope scores before and after using VR, both in healthy children and in hospitalized children. VR is a promising technique for cognitive diversion and psychological rehabilitation support for patients with cancer [46], particularly when they experience boredom during hospitalization following treatment [47,48]. Such computerized technologies are especially appealing to this age group, which, in turn, increases their enthusiasm [46]. For example, the results revealed no significant difference in pleasure and travel expectations between the hospitalized and healthy children. We infer that this finding suggests that both hospitalized and healthy children experienced pleasure when using VR because it was a novel experience for them and sparked their expectations about traveling. Although some participants in the hospitalized children group had prior experience using VR, there were no significant differences in hope and travel expectations between those who had used VR before and those who had not. Therefore, we suggest that health care workers shift their focus from health to overall well-being [49] and incorporate immersive VR experiences into pediatric care as a form of distraction therapy. This approach can help children cope with long-term hospitalization and enhance their emotional well-being.

### Study Limitations and Recommendations for Future Research

Some limitations of this study should be noted. First, the study examined differences between healthy children and hospitalized children with leukemia aged 7‐18 years. Therefore, the results are applicable only to hospitalized children with leukemia in the midstage of treatment with stable vital signs and not to those with different medical conditions or from other age groups. Future studies should investigate the impact of VR apps on other age groups and medical conditions. Second, the VR experience content used in this research was primarily based on tourist attractions. Future studies could explore different VR content and experiences. Third, we acknowledge the limitations of ECG in differentiating specific emotional responses, as an increased heart rate can be associated with multiple emotions such as joy, surprise, or satisfaction. This is why we combined questionnaires to enhance the interpretation of emotional responses. Future studies could use more precise psychophysiological methods, such as functional magnetic resonance imaging, to improve the accuracy of emotional response assessments.

### Conclusions

With the advancement of emerging technologies, this study aimed to evaluate the pleasurable experience of VR and its impact on hospitalized children’s travel expectations and hope by using ECG and questionnaires. We examined the differences between healthy and hospitalized children in their perceptions of pleasure and hope to provide future scholars with a reference for research on hospitalized children. In this study, VR provides hospitalized children with a safe space and a more immersive way to explore the world. It also provides another way for children to experience the world in addition to books and photographs, and it gives children more opportunities to learn about the world. Through self-learning and adaptation, children can find ways to deal with their emotions and relieve their psychological pressure. VR apps are considered an accessible, low-risk, and cost-effective innovative approach for children with leukemia, particularly during repeated waves of the epidemic, as they minimize the risk of cross-infection [50].

## Abbreviations

ANS: autonomic nervous system
ECG: electrocardiography
HF_nu: high-frequency normalized unit
LF: low-frequency power
PNS: parasympathetic nervous system
SDNN: SD of all normal-to-normal intervals
SNS: sympathetic nervous system
VR: virtual reality
